# Preclinical Cold Atmospheric Plasma Cancer Treatment

**DOI:** 10.3390/cancers14143461

**Published:** 2022-07-16

**Authors:** Ruby Limanowski, Dayun Yan, Lin Li, Michael Keidar

**Affiliations:** 1 Department of Biomedical Engineering, George Washington University, Washington, DC 20052, USA; rubyliman@gwmail.gwu.edu; 2 Department of Mechanical and Aerospace Engineering, George Washington University, Washington, DC 20052, USA; lilin@email.gwu.edu

**Keywords:** cold atmospheric plasma, cancer treatment, anti-tumor therapy, reactive species, non-invasive therapy, redox medicine, drug sensitization

## Abstract

**Simple Summary:**

Cold atmospheric plasma (CAP) is generated in a rapid yet low-energy input streamer-discharge process at atmospheric pressure conditions. CAP is an ionized gas with a low ionization level and plenty of reactive species and radicals. These reactive components, and their near-room temperature nature, make CAP a powerful tool in medical applications, particularly cancer therapy. Here, we summarized the latest development and status of preclinical applications of CAP in cancer therapy, which may guide further clinical studies of CAP-based cancer therapy.

**Abstract:**

CAP is an ionized gas generated under atmospheric pressure conditions. Due to its reactive chemical components and near-room temperature nature, CAP has promising applications in diverse branches of medicine, including microorganism sterilization, biofilm inactivation, wound healing, and cancer therapy. Currently, hundreds of in vitro demonstrations of CAP-based cancer treatments have been reported. However, preclinical studies, particularly in vivo studies, are pivotal to achieving a final clinical application. Here, we comprehensively introduced the research status of the preclinical usage of CAP in cancer treatment, by primarily focusing on the in vivo studies over the past decade. We summarized the primary research strategies in preclinical and clinical studies, including transdermal CAP treatment, post-surgical CAP treatment, CAP-activated solutions treatment, and sensitization treatment to drugs. Finally, the underlying mechanism was discussed based on the latest understanding.

## 1. CAP and Plasma Sources

CAP has been widely used in several branches of medicine, including wound healing, microorganism sterilization, biofilm inactivation, and cancer therapy [1,2,3]. CAP is an ionized gas composed of reactive compounds such as reactive oxygen species (ROS) and reactive nitrogen species (RNS) [4,5], and is designed to work under atmospheric pressure at a near room temperature [6].

The physical foundation to generate CAP is briefly illustrated here. We take the atmospheric pressure plasma jet (APPJ) as an example, which has been more widely used in plasma medicine than any other sources [7]. Typical CAP generations usually rely on a specific ionization process, namely “positive streamer propagation,” as a kind of ionization wave [8,9]. The positive streamer propagation starts near the anode, where the seed electrons are attracted. During their movements, electrons collide with other particles. As the electric field near the anodes reaches an adequately high level, several electrons are accelerated sufficiently to ionize the particles, resulting in more electrons, namely an “avalanche,” while other electrons with lower velocities may only excite the particles. The ionization wavefront is where the “avalanche” occurs. The wavefront is the luminous region due to the photon emissions from the excited atoms or other particles, which is also known as the “plasma bullet” [8,9]. During the streamer propagation in the open air with a noble gas environment such as helium, hundreds of chemical reactions occur simultaneously, each with a unique but dynamic reaction rate [10]. These chemical reactions thus generate ROS/RNS. In many cases, CAP sources are powered with alternating current (AC) to ensure a continuous generation in the open air [11].

**Figure 1:**
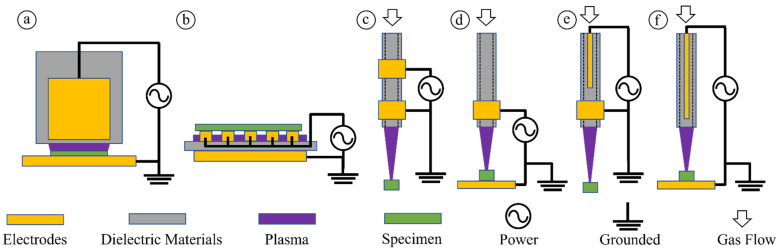
Typical CAP sources used in plasma medicine. Type a. Volume DBD. Type b. Surface DBD. Type c. Two-ring electrodes APPJ. Type d. Plasma torch using ring electrode. Type e. One central electrode-one ring electrode APPJ. Type f. Plasma torch using a central electrode.

CAP sources, such as dielectric barrier discharge (DBD), APPJ, and plasma torch, are the foundation for plasma applications [12,13]. Six of the most commonly used CAP sources are shown in Figure 1. AC is a typical power supply for these CAP sources [14]. Type a and type b are DBD-style devices. DBD generates plasma between two electrodes powered by radiofrequency (RF) discharge voltage, usually around the kV scale. Multiple streamer propagations developed between the electrodes in each discharge cycle, and each streamer path is called a “filament” [15]. Although the horizontal spatial distribution of filaments is random and dynamic, the distribution is uniform when the two electrode surfaces are parallel, and the dielectric barrier material properties are uniform. Therefore, the plasma generated from DBD can cover a large area. On the other hand, APPJ (type c and type e) and plasma torch (type d and type f) are more focused on tools that can deliver ROS/RNS on a small size target more accurately as a single track of streamer propagation. An APPJ generator requires a hollow cathode to allow the streamer to pass through and reach the target below the cathode. However, the plasma torch is the standard model for streamer propagation between two electrodes. In addition to these typical CAP sources, some new sources have recently been developed and used in preclinical studies. One example is a non-invasive and non-thermally operated electrosurgical plasma source [16,17,18].

## 2. General Picture of In Vitro Studies

To date, the promising anti-cancer performance of CAP treatment in vitro has been extensively demonstrated in dozens of cancer types, including skin, breast, colorectal, brain, lung, cervical, head and neck cancer [3,7]. Plenty of reviews and articles have been published, most of them focused on in vitro studies and corresponding conclusions [19,20,21]. Several basic cellular responses have been repeatedly observed in the publications listed in Table 1. These basic cellular responses build the foundation for understanding the anti-cancer effect of CAP treatment in vitro and address some in vivo observations.

Together, some general conclusions can be summarized here. (1) Reactive species play a critical role in the liquid phase-based experimental setting [39]. Apoptosis, necrosis, and autophagy are the main cellular death approaches following CAP treatment with an adequately large dose [40]. (2) Physical factors, particularly electromagnetic effects from plasma, may exert a clear impact on cells, such as bacteria and mammalian cells [41,42]. (3) A noticeable rise in intracellular ROS is a pivotal cellular response to CAP treatment, which further triggers downstream cellular damage, including DNA damage, mitochondrial damage, cellular membrane damage, and cell death [1]. (4) Aqueous environment, such as a medium layer, plays a pivotal role in facilitating the transition of short-lived reactive species in the gas phase into long-lived reactive species in the liquid phase [43,44,45]. For in vitro studies, a medium layer is necessary for experimental design and is responsible for most observed cellular responses after CAP treatment, particularly for the cases involving CAP-treated solutions or media [40,46]. (5) CAP shows selective killing effect on cancer cell lines compared to their counterpart normal cell lines in many cases [47].

## 3. Direct CAP Treatment In Vivo

Like most medical studies, the conclusions obtained from in vitro studies cannot be easily used to directly predict the performance of in vivo studies. For example, the relatively dry skin barrier between plasma and targeted cancerous tissues or cells under the skin is quite different from the commonly accepted experimental conditions in vitro. The in vitro environment mainly involves a relatively thick medium layer to facilitate the transition of some short-lived reactive species in gas phase to long-lived reactive species in liquid phase. Moreover, both long-lived and short-lived reactive species will have complex reactions at this gas/liquid interface. For plasma medicine, in vivo studies play a cornerstone role before CAP can be used in clinical therapy [48,49,50]. More importantly, in vivo studies directly assess the CAP treatment’s safety on tissues and animals, such as carcinogenicity [51]. In this review, our preclinical studies’ discussion will be just limited to in vivo studies.

Compared to the abundant in vitro investigations, in vivo studies have gradually become the main approach to discovering novel tissue responses to CAP treatment. Animal models’ design directly determines the use of CAP in the in vivo studies. So far, three types of animal models have been widely used to demonstrate the anti-tumor efficacy of CAP treatment: subcutaneous model, intraperitoneal model, and orthotopic model [52]. To date, most of CAP’s anti-tumor capability was demonstrated by using subcutaneous models.

**Figure 2:**
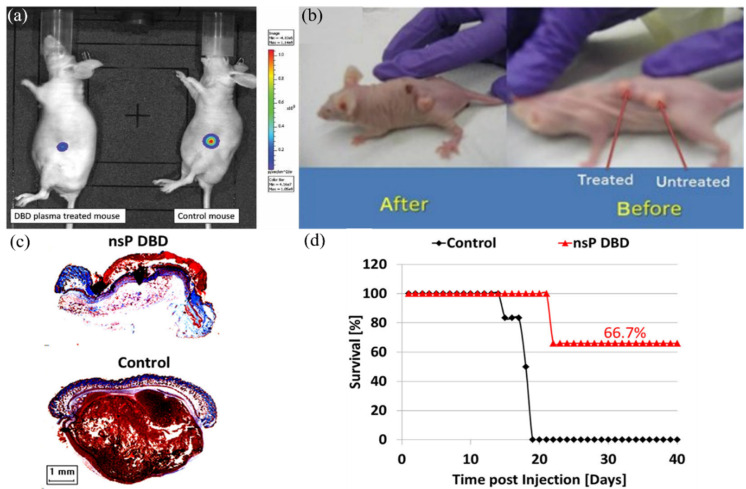
Some representative anti-tumor demonstrations use subcutaneous models. (**a**) U87 xenografted nude mice mouse was irradiated (6 min) by a DBD device for 5 consecutive days. Bioluminescence imaging (BLI) was used to quantify tumor growth and size. Reprinted with permission from Ref. [53]. 2010, Wiley. (**b**) Typical image of mice with three subcutaneous Bladder xenografted tumors before and 24 h after APPJ treatment. [49]. (**c**) nsp DBD treatment strongly eradiated melanoma tumors in mice. Trichrome staining of DBD treated tumor (top) and control tumor (bottom). (**d**) Survival for DBD treated tumors (red) and untreated tumors in control (black) as a function of time post-injection. [48].

Subcutaneous models provided the earliest and the most apparent demonstration for the feasibility of using CAP as an anti-tumor modality. The earliest in vivo works were demonstrated by Marc Vandamme, et al. and Michael Keidar, et al. between 2010–2011 (Figure 2a,b). They used a glioblastoma U87MG xenograft mouse model and bladder xenograft tumor model to test a CAP treatment’s in vivo efficacy for just a few minutes, respectively. The two pioneering research articles demonstrated a drastic tumor volume reduction of more than 50% after floating electrode DBD treatment and APPJ treatment [49,53]. Correspondingly, the survival length of mice strongly increased by more than 60% in the two models [49,53]. These two works also first tested the safety of using CAP in animal studies. Results did not show any toxic side effects and potential physical damage from plasma.

Due to the subcutaneous nature of melanomas, it has become one of the more promising candidates for CAP-based cancer therapy. Many studies have been performed on melanoma models [7]. As shown in Figure 2c, a nanosecond pulsed DBD (nsP DBD) completely eradicated the xenografted melanoma tumor in mice after direct treatment on the skin above the melanoma. Histology of an nsP DBD treated tumor showed a typical red skin staining without tumor tissue below the epithelium. Correspondingly, the survival rate of mice increased from 0% to 66.7% 20–40 days succeeding the nsp DBD treatment (Figure 2d) [48].

Similar trends have been repeatedly observed in a series of following studies. Table 2 lists representative in vivo anti-tumor demonstrations (2010–2018) on subcutaneous xenograft tumors in mice. In the subcutaneous model, CAP treatment was mainly carried out by treating the skin above tumorous tissues. In such a setting, the effective factor, either chemical or physical factors in CAP, must penetrate the skin barrier and further trigger biological pathways to inhibit tumorous growth, therefore providing CAP treatment as a potential non-invasive anti-tumor modality. Among these studies, some general trends have been observed. First, a treatment just above the skin could strongly inhibit the growth of tumors and significantly extend the life length of mice [53]. Second, a fractionated, multi-time consecutive treatment may generate a much better therapeutic effect than a single but long treatment, which may be due to the long-term anti-tumor effect of CAP treatment [54]. A multi-time consecutive treatment may consecutively trigger these long-term anti-tumor effects, such as an immune response.

## 4. CAP-Activated Solutions (PAS) and In Vivo Application

Direct CAP treatment is based on the touch of bulk plasma with or near a target. In contrast, indirect treatment is based on the CAP-activated (treated, stimulated) solutions to affect the growth of cancer cells or tissues. Over the past decade, CAP-activated solutions (PAS) have shown promising applications in cancer treatment in vitro and in vivo [46]. Once PAS is made, it can be stored for days or weeks and used in cases without a CAP source [64,65]. PAS can be employed to inhibit the growth of tumorous tissue by subcutaneous or intraperitoneal injection in mice [66]. Moreover, PAS can be used in the lavage of patients suffering from peritoneal carcinomatosis adjuvant to standard chemotherapy [67,68]. Typically, PAS is made by treating biologically adaptable solutions such as a medium or phosphate-buffered saline (PBS) using APPJ or DBD above a solution’s surface [69,70,71]. PAS can also be made by underwater discharge in solutions [72]. PAS can be synergistically used to enhance the therapeutic effects of chemotherapy drugs and other chemicals [73,74,75]. PAS selectively kills colon, lung, cervical, bladder, melanoma, and breast cancer cells in vitro [76,77,78,79,80]. Preliminary studies on immuno-deficient nude mice by oral lavage treatment of CAP-activated water did not find lethal effects and acute toxicity [81,82]. Furthermore, the mice had no significant changes, including body weight, survival status, organ coefficient, function, and tissue structure of heart, liver, spleen, lung, and kidney [81,82].

Several in vivo studies present the potential use of PAS in cancer therapy. Several representative studies were introduced here. Fumi Utsumi et al. first achieved an evident anti-tumor efficacy in mice by injecting CAP-activated Ringer’s lactate into subcutaneous tumors grown from the xenografted chemical-resistant ovarian cancer cells [83]. Compared with PBS, Ringer’s lactate solution is closer to the translation solution, which could be used as a clinical modality [84,85,86]. Ringer’s lactate solution contains sodium chloride, potassium chloride, calcium chloride, sodium lactate, and sodium bicarbonate. PAS can also be used for intraperitoneally xenografted tumor models. Shigeomi Takeda et al. demonstrated that PAS effectively decreased the peritoneal metastatic nodules by 60% in mice without causing adverse events [67]. Similar anti-tumor performance by injection of PAS in vivo have been observed in other subsequent studies [80,87].

**Figure 3:**
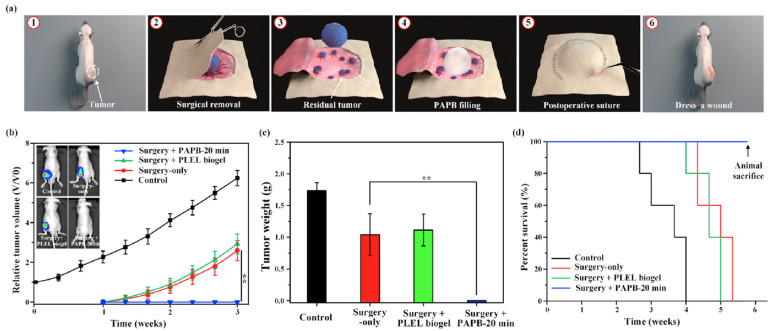
Anti-tumor performance of a plasma-activated PLEL biogel (PAPB) on residual tumor after surgical removal. (**a**) Schematic illustration of post-operative PAPB treatment. (**b**) Whole-body bioluminescence imaging and tumor growth in mice: control, surgical removal-only, surgical removal + PLEL (thermosensitive biogel), and surgical removal + PAPB treatment. (**c**) Weight of excised tumors. (**d**) Survival of tumor-bearing mice (*n* = 5, ** *p* < 0.01). Reprinted with permission from Ref. [88]. 2021, Elsevier Ltd.

Recently, a novel strategy to use PAS has been demonstrated. Post-surgical residual tumor tissues or cells are the primary cause of relapse and progression of cancer post-surgery. A fillable plasma-activated biogel was made on a thermosensitive biogel, (poly-dl-lactide)-(poly-ethylene-glycol)-(poly-dl-lactide), PLEL, with the aid of PAS for local post-surgical removal of tumors in mice models [88]. Preliminary in vivo data demonstrated that the plasma-activated PLEL biogel (PAPB) entirely eliminated residual in situ tumor tissue recurrence after a removal surgery without showing evident systemic toxicity (Figure 3). More attractively, PAPB allowed a slow release of ROS. Altogether, this study provided a solid foundation to use PAS in local post-operative cancer treatments [88].

## 5. Abscopal Effect

**Figure 4:**
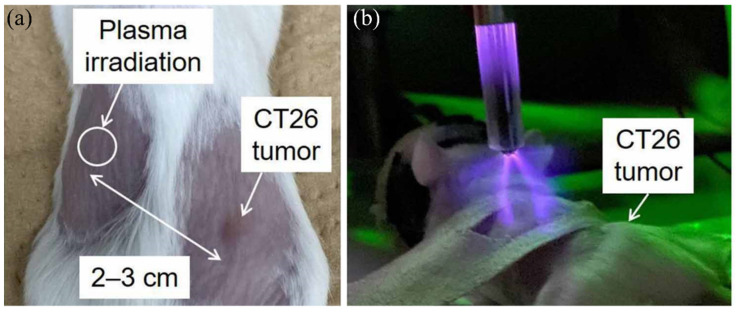
Anti-tumor abscopal effects in mice induced by a CAP treatment on normal tissue. (**a**) CAP irradiation site and murine colorectal carcinoma CT26 tumor on day 6. (**b**) CAP treatment on a normal tissue site can inhibit the nearby tumor’s growth [90].

An anti-tumor abscopal effect was rarely observed in plasma medicine and radiotherapy. To date, only two examples were reported in 2017 and 2022, providing a novel approach to using CAP in cancer therapy. Compared to previous studies, these two studies demonstrated that the tumor growth at a non-treated site on a mouse could be suppressed by a CAP treatment on another nearby tumor site or even by a CAP treatment on the health tissue site on the same mouse (Figure 4). Moreover, these surprising phenomena appeared just one day post the treatment [89]. Particularly, when CAP treatment was performed on the skin above the healthy tissue of the left limb, the abscopal effect was only significant for the mice with small tumors of the right limb [90]. Fundamentally, the abscopal effect triggered by a CAP treatment on the skin above normal tissue was comparable to the anti-tumor efficacy of a direct CAP treatment on the skin above tumorous tissue.

**Figure 5:**
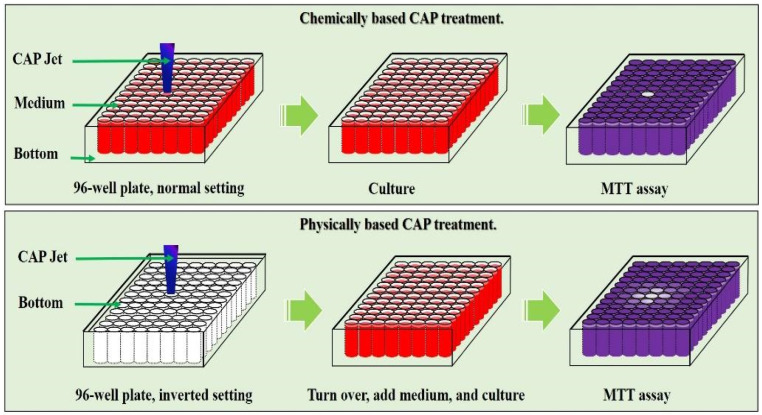
Physical factors in CAP can affect a much bigger area than the plasma contacting area on samples. A representative illustration of chemical factors and physical factors in CAP and their killing effect on cancer cells in vitro [41].

Due to the very limited data from these two studies, the underlying mechanism is entirely unknown. The authors explained that the innate immune response in vivo might trigger such a rapid abscopal effect after observing the production of inflammatory cytokines (IFN -γ) from splenocytes post CAP treatment [89]. Here, based on recent discoveries of the physically based CAP treatment, we proposed that physical factors in CAP may explain these abscopal effects. Physical factors in CAP, likely mainly electromagnetic (EM) effect, can affect the target with an area much larger than the plasma size or the contacting area between plasma and target. As shown in Figure 5, physically based treatment can affect an area much larger than chemically based treatment because the EM effect can penetrate the dielectric physical barrier between every well on a 96-well plate [41]. In our recent study, an APPJ’s tip was less than 1 mm; however, the EM effect generated from APPJ could affect an area with a diameter of at least 2 cm [91]. As shown in Figure 4a, the abscopal effect affects the tumor at a distance of 2–3 cm. The physical effect of CAP treatment is much more complex than its chemical effect in terms of spatial distributions and anisotropic effect, which may cause a series of novel biological effects.

## 6. Sensitization of Tumor to Drugs

The primary rationale for using CAP in cancer treatment is to achieve a direct killing effect on tumor or cancer cells in vitro and in vivo. Recently, a novel rationale for using CAP has caught attention, which is focusing on using CAP to sensitize cancer cells or tissues to the existing chemotherapy, particularly the cytotoxicity of drugs. Unlike previous studies focusing on the chemically based sensitization of cancer cells to some drugs, this new approach focused on using the physical factors, mainly EM effect or EM emission from CAP, to affect tumors in depth rather than just to affect the subcutaneous models. So far, it is still unclear the effective EM frequency range to cause these EM effects. Thus, the current studies focused on demonstrating the sensitization capability of CAP treatment on brain cancer models such as glioblastoma in mice brains. The underlying mechanism is entirely unknown at the current stage [92].

**Figure 6:**
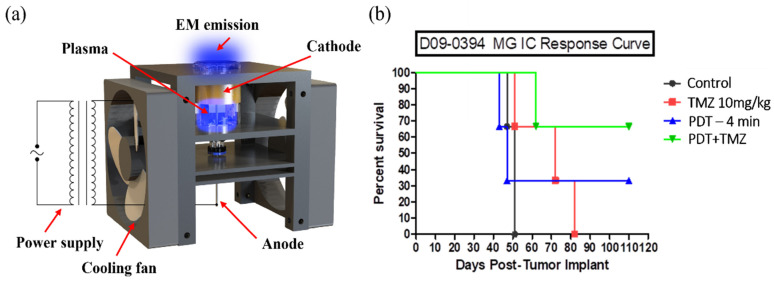
Sensitization of brain cancer cells to temozolomide (TMZ) by a cold plasma discharge tube (PDT). (**a**) Basic structure of PDT source. (**b**) Mouse survival rate curve. Reprinted with permission from Ref. [96]. 2022, American Chemical Society.

Glioblastoma (GBM) is one of the most aggressive brain cancers. GBM is also highly resistant to treatment [93,94]. Temozolomide (TMZ) is a widely used FDA-approved alkylating chemotherapy agent, particularly for high-grade malignant GBM treatment [94]. Recently, two studies demonstrated that just a CAP treatment during a mouse’s brain neurosurgery could achieve a noticeable enhanced therapeutic efficacy of TMZ. First, a single APPJ treatment on the mouse’s head could sensitize brain tumors in the skull to the cytotoxicity of GBM [95]. Another example was using a helium radial cold plasma discharge tube (PDT) as a tunable EM emission source, which only allowed the EM effect of CAP to affect targets because all chemical factors have been blocked in PDT (Figure 6a). PDT selectively increased the cytotoxicity of TMZ on two glioblastoma cell lines A172 and U87MG, compared to the standard astrocyte cell line hTERT/E6/E7 to some extent in vitro [96]. More attractively, preliminary in vivo studies demonstrated a drastically improved mean survival day of patient-derived xenografted glioblastoma mice models by 100% compared to the control group (Figure 6b). PDT was independent of continuous gas supply; thus, it has the potential to be a portable and small CAP source. Together, these two studies demonstrated that the EM effect in CAP could penetrate the skin and the skull, providing an unprecedented vision for further CAP-based cancer therapy.

## 7. Clinical Anti-Tumor Trials

The clinical use of CAP as an anti-tumor modality is an ultimate goal in plasma medicine. Unfortunately, CAP’s clinical tests in cancer therapy are still quite rare. A few examples were illustrated here, which provide critical clues to guide the use of CAP in therapeutic advances. In 2015, a private company, US Medical Innovation (USMI), carried out a clinical trial on stage IV metastatic colon cancer at Baton Rouge General Medical Center in Baton Rouge, Louisiana, USA. CAP treatment was performed on the post-surgical tissue to kill potential residual cancer cells after a removal surgery, and no relapse and progression of cancer occurred in patients. The trial also tested the safety of CAP treatment [97]. In Germany, CAP treatment was performed on 12 patients with advanced squamous cell carcinoma of the head and neck [98]. CAP was used to decontaminate infected cancer ulcerations in this trial. It is found that CAP treatment generated positive effects in patients, including a decreased request for pain medication and a reduction of typical fetid odor and microbial load [98]. In some cases, superficial partial remission of tumor and even wound healing of the infected ulcerations have been observed [98].

**Figure 7:**
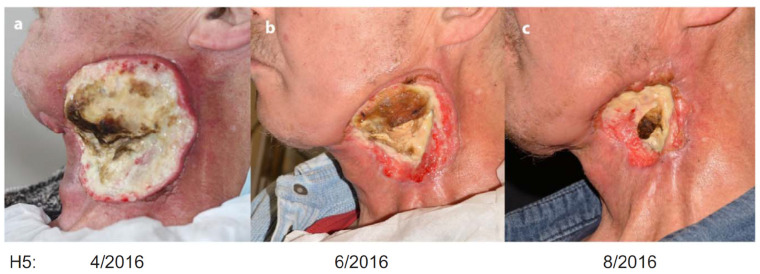
The clinical effect of CAP treatment on a patient (H5) with locally advanced head and neck cancer. Reprinted with permission from Ref. [99]. 2017, Elsevier GmbH. The patient’s therapeutic effect was recorded in April/2016 (**a**), June/2016 (**b**), and August/2016 (**c**), respectively.

In 2017, USMI used Canady Helios Cold Plasma and Hybrid Plasma Scalpels in the clinical liver resection to remove and selectively kill liver tumor cells [97]. In the same year, there was another impactful treatment performed by Metelmann et al. [99]. Their trial enrolled six patients with local advanced (pT4) squamous cell carcinoma of the oropharynx with open infected ulcerations. Six patients were treated by an APPJ in a cycle of three single applications within a week, each followed by an intermittence of another week [99]. As shown in Figure 7, CAP treatment noticeably improved the therapeutic effect of this locally advanced head and neck cancer. CAP treatment not only improved patients’ social functions, but also caused a reduction in odor and pain medication requirements [99]. In addition, partial remission in two patients has been observed, and the incisional biopsies found a moderate level of apoptotic tumor cells and a desmoplastic reaction in the connective tissue [99]. These clinical trials also strongly suggested that CAP treatment’s widely observed wound healing capability will play a critical supporting role in cancer therapy [100]. In May 2022, USMI presented the results of a two-year follow up phase I clinical trial of Canady Helios Cold Atmospheric Plasma (CHCP) treatment for patients with advanced stage IV metastatic and recurrent solid tumors at the Biannual Conference of the Israeli Society of Surgical Oncology. Twenty patients were recruited from U.S. and Israel. Patients received intra-operative CHCP treatment at the operative site after removing the tumor. The primary endpoint was safety [101]. Together, the existing clinical trial suggests that CAP treatment can be a powerful supplemental tool to improve the current surgery and chemotherapy efficacy.

## 8. Mechanism Discussion

Due to the complex nature of CAP and tissues, the anti-tumor mechanism of CAP in vivo is an open question. Reactive species, particularly ROS, have been widely regarded as the main factors in CAP to cause cellular damage and ultimate cell death in vitro [5,102]. The strong rise of ROS in the tumor tissue after CAP treatment has also been observed [103]. This cellular response may be due to the transdermal diffusion of reactive species [104]. Because of the complexity of mammalian skin, the transdermal diffusion process was mainly studied using skin substitutes such as agarose gels [105]. For example, it is found that H_2_O_2_ and NO_2_^−^ could be slowly (30 min) transported through an agarose gel with a thickness of 1.5 mm [106]. Furthermore, the transportation of reactive species through an agarose gel is highly dependent on gel thickness [107]. A 10-mm thick gap of gas between the agarose gel and CAP could inhibit reactive species’ transmembrane diffusion [107]. Recently, via using contact- and marker-independent Raman microscopy, it was found that the APPJ can penetrate the basal cell layer of a cervical epithelium sample with a depth of roughly 270 μm [108].

The physical pathways in the skin necessary for the transdermal diffusion of reactive species are still unknown. Possible pathway candidates may include hair follicles, microneedles, electroporation, or other transcellular and intracellular routes [109]. Recently, the transdermal transportation of reactive species across animal skin, such as mouse and pig skin, have been studied. An air CAP source was used to treat mouse skin with a thickness of 0.75 mm, and the authors did not observe the formation of H_2_O_2_ and NO_2_^−^/NO_3_^−^ in the deionized water underneath the skin [110]. Nevertheless, the authors did observe the transdermal diffusion of NO_2_^−^/NO_3_^−^ in the CAP-activated deionized water across the mouse skin [110]. The transdermal diffusion of RONS across pig skin has been observed under specific operational conditions, such as discharge frequency [111,112]. A recent study found that a 300-ns 50-kV/cm pulsed electric field increased the transdermal diffusion of RONS across a pig skin model [113]. Altogether, these preliminary investigations suggest that the transdermal diffusion of reactive species underneath a millimeter-level thick tissue-mimic film is possible after CAP treatment.

**Figure 8:**
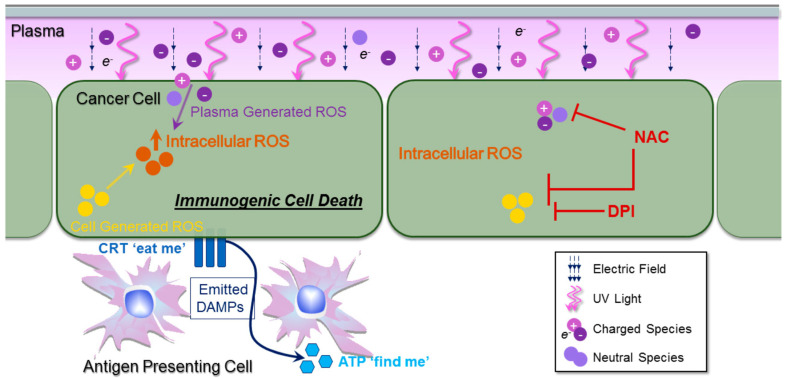
A CAP-based immunogenic cell death (ICD) in vivo model. Both chemical and physical factors in CAP may affect cellular functions. ROS, RNS, or other chemical factors may mainly contribute to eliciting ICD. CAP triggers the DAMP signals, such as ecto-CRT and ATP secretion. ATP acts as a “find me” DAMP signal to recruit immune cells, and surface-exposed CRT serves as an “eat me” signal. Diphenyleneiodonium (DPI) could inhibit cellular ROS production. NAC could scavenge ROS from different sources [117].

How do the CAP-originated or secondary reactive species affect subcutaneous tumor tissues? The widely observed subcutaneous anti-tumor effect may affect the tissues by using the direct killing effect from these RONS or due to the activation of an immune response triggered by CAP treatment. The development of CAP in combination with cancer immunotherapeutics has received growing attention recently. One rationale is that CAP may activate the immune system to attack tumorous tissue by reactive species or other factors [114,115]. These immune responses have been named as immunogenic cell death (ICD) [32,63,89,114,116,117,118]. One early representative observation found that macrophages could be activated in vitro by nsp DBD [116]. Some studies found that CAP could trigger cancer cells to emit signals known as damage-associated molecular patterns (DAMP), which may attract and stimulate local immune cells [119]. As shown in Figure 8, DAMP include at least two types of signals: a “find me” signal such as ATP and an “eat me” signal such as ecto-CRT [117]. Some studies observed increasing exposure of CRT on a cancer cells’ surface [117,120]. Moreover, specific expression of molecular pattern signals ATP has been observed and were believed to further trigger immunogenic attack on cancer cells [117].

CAP causes ICD far beyond these approaches. For example, the injection of the CAP-treated CT26 colorectal cancer cells in mice caused a noticeable growth inhibition in the tumor compared to injecting the CT26 cells without CAP treatment [120]. In other words, the CAP-treated cancer cells can be used as a whole-cell vaccine to elicit protective immunity in at least CT26 colorectal tumor mouse models [63]. Similar research strategies have been reported recently in other studies [121]. Furthermore, the synergistic use of CAP with vaccination enhanced the cancer-specific T-cell responses as well [63,120]. In short, immunogenic cell death may be a core process to understand in vivo anti-tumor performance of a CAP treatment.

Other factors in CAP may also trigger an immune response. For example, a strong (micromolar level) cell-based H_2_O_2_ generation has been observed during and following a CAP treatment using APPJ in vitro [33,122,123]. H_2_O_2_ is a second messenger to activate lymphocyte [124,125]. Micromole levels of H_2_O_2_ could rapidly activate the transcription factor NF-κB and early gene expression of interleukin-2 (IL-2) [124]. Short-lived ROS such as superoxide or single oxygen may activate cancerous cells to generate H_2_O_2_ [33]. Tumorous tissues may generate plenty of H_2_O_2_ after CAP treatment in vivo, and the CAP-originated H_2_O_2_ does not directly touch these tissues, which explains the strong rise of subcutaneous ROS in the CAP-treated mice [103]. If similar phenomena also occur in vivo, the CAP-affected tissue may become a target for the immune system’s attack.

Nevertheless, the conclusion may not simply be used to explain many observations in vivo. Physical factors in CAP may play critical roles when CAP source is used to directly treat tumor tissues or even just using CAP source to affect the subcutaneous tissue with skin as the barrier [126]. For in vitro studies, a liquid layer always covers the cells due to the experimental setting. As a result, physical factors, thermal, UV, or other EM effects, have been entirely or largely blocked by the liquid layer [40]. Thus, physical factors’ effect has not been observed until the recent direct demonstration of a strong anti-cancer effect using physically based CAP treatment. Physical factors, likely mainly the EM effect generated in CAP, have several novel features compared with chemical factors such as ROS and RNS in CAP. First, physical factors in CAP can penetrate the dielectric barrier (~1 mm) and air gap (~8 mm) to affect cells, which is consistent with the widely observed non-invasive nature of the transdermal capability of CAP treatment on the skin above tumor tissues [36,91]. Second, physical factors in CAP cause strong necrosis [36,41,127]. Necrosis will trigger inflammation and other immune responses in vivo, which may explain some immune responses in many in vivo studies.

**Figure 9:**
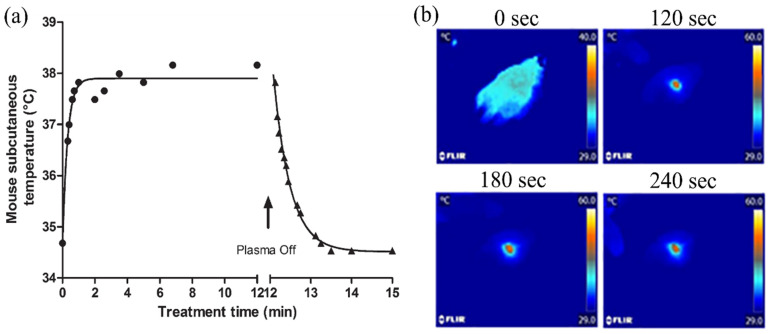
Thermal effect of CAP treatment on the mouse. (**a**) Evolution of mouse subcutaneous temperature during and after DBD treatment. Reprinted with permission from Ref. [53]. 2010, Wiley. (**b**) Temperature distribution infrared imaging on the APPJ-treated (4 min) mouse by a thermal visor [50].

Furthermore, the thermal effect of CAP treatment cannot be simply ignored. For example, in the earliest in vivo demonstration (Figure 9a), the subcutaneous temperature of a mouse after the treatment was close to the mouse’s body temperature [53]. However, in another example (Figure 9b), the highest temperature in the treated mouse’s skin was at least 50 °C after 4 min of treatment [50]. Clearly, the heating effect in this case and other similar cases must be considered when the anti-tumor mechanism is analyzed. Thus, the temperature data or the thermal effect in CAP treatment must be provided or be considered in future in vivo studies. The naming of “CAP” cannot naturally guarantee the plasma is actually “cold” or “nonthermal”. For example, some so-called “self-organized” patterns of plasma have a temperature of hundreds °C [128]. Strictly speaking, these “hot” CAP sources cannot be regarded as cold plasma sources.

## 9. Conclusions

Altogether, it is promising that CAP will play a unique role as a novel, self-adaptive anti-tumor weapon. In a clinic, however, CAP cannot be used to quickly remove a tumor tissue, which may be the largest limitation of its anti-tumor performance compared to surgical approaches. To date, in clinical trials, CAP has been used either by treating the potential residual tumor tissues post-surgical removal or by directly treating tumor sites to improve other therapeutic modalities’ efficacy. Currently, CAP is more like a surgery-assistant tool in cancer therapy.

Further clinical applications may be beyond this vision. Over the past decade, three strategies to use CAP have been proposed and demonstrated in preclinical studies. First, the direct killing effect of CAP on tumors by either the non-invasive transdermal diffusion of reactive species or by the immunogenic cell death of cancer cells after CAP treatment. This strategy is suitable for subcutaneous tumors, such as melanoma and neck and head cancer. Second, for the tumors in depth, such as intraperitoneally tumor models, PAS can be injected into deep tissue to inhibit tumor growth. Lastly, a novel strategy is the sensitization of cancer cells to the cytotoxicity of chemotherapeutic drugs, either by reactive species or by physical factors such as EM emission from CAP sources. Generally, these biological responses of CAP may be not only due to chemical factors such as reactive species but also physical factors such as EM and thermal effects.

## Figures and Tables

**Table 1:** Basic cancer cellular responses of CAP treatment in vitro.

Ref	Cancer Cellular Response	Years
[22]	Apoptosis	2004
[23]	Growth Inhibition	2007
[24]	Cytoskeletal Damage	2009
[25]	Selective Cell Death	2010
[26]	Cell Cycle Arrest	2010
[27]	Nuclear and DNA Damage	2010
[27]	Mitochondrial Damage	2010
[28]	Rise of Intracellular ROS	2011
[29]	Chemically-based Sensitization to Drugs	2013
[30]	Selective Rise of Intracellular ROS	2013
[31]	Senescence	2013
[32]	Immunogenic Cell Death	2015
[33]	Cell-based H_2_O_2_ Generation	2017
[34]	Autophagy-associated Cell Death	2017
[35]	Activation Phenomena	2018
[36]	Physically-triggered Necrosis	2020
[37]	Pyroptosis	2020
[38]	Physically-based Sensitization to Drugs	2021

**Table 2:** Representative in vivo demonstrations on subcutaneous xenografted tumor models (2010–2018).

Ref	Years	Tumor Types	Tumor Size	Survival Rate	Tumor Diagnostics
[53]	2010	Glioblastoma	Decreased	N/A	Bioluminescence imaging
[49]	2010	Bladder cancer	Decreased	Increased	Tissue size measurement
[54]	2011	Glioblastoma	Decreased	Increased	Bioluminescence imaging
[55]	2012	Pancreatic carcinoma	Decreased	N/A	Bioluminescence imaging
[56]	2012	Glioblastoma	Decreased	N/A	Bioluminescence imaging
[57]	2013	Neuroblastoma	Decreased	Increased	Tissue size measurement
[58]	2014	Melanoma	Decreased	N/A	Tissue size measurement
[59]	2014	Head and neck cancer	Decreased	N/A	Tissue size measurement
[48]	2015	Melanoma	Decreased	Increased	Tissue size measurement
[60]	2015	Endometrioid adenocarcinoma	Decreased	N/A	Tissue size measurement
[61]	2016	Glioblastoma	Decreased	N/A	Tissue size measurement
[62]	2016	Breast cancer	Decreased	N/A	Tissue size measurement
[50]	2017	Melanoma	Decreased	N/A	Bioluminescence imaging
[63]	2018	Colorectal tumor	Decreased	N/A	Tissue size measurement

## Abbreviations

AC	Alternating Current
APPJ	Atmospheric Pressure Plasma Jet
ATP	Adenosine Triphosphate
CAP	Cold Atmospheric Plasma
CHCP	Canady Helios Cold Atmospheric Plasma
CRT	Calreticulin
DAMP	Damage-Associated Molecular Patterns
DBD	Dielectric Barrier Discharge
DPI	Diphenylenyleneiodonium
EM	Electromagnetic
ICD	Immunogenic Cell Death
IFN	Inflammatory Cytokines
IL	Interleukin
nsP	Nanosecond Pulsed
PAS	CAP-Activated Solutions
PAPB	PLEL Biogel
PBS	Phosphate-Buffered Saline
PDT	Cold Plasma Discharge Tube
PLEL	(Poly-DL-Lactide)-(Poly-Ethylene-Glycol)-(Poly-DL-Lactide)
RF	Radiofrequency
ROS	Reactive Oxygen Species
RNS	Reactive Nitrogen Species
TMZ	Temozolomide
USMI	US Medical Innovation
